# Estudio comparativo de cuatro analizadores diferentes para la evaluación prenatal del riesgo de trisomía 21

**DOI:** 10.1515/almed-2025-0149

**Published:** 2025-11-11

**Authors:** Natalia García-Simón, Cristina Martínez-Payo, Francisco A. Bernabeu-Andreu, Encarnación Donoso-Navarro

**Affiliations:** Laboratorio Bioquímica Clínica-Análisis Clínicos del Hospital Puerta de Hierro Majadahonda, Majadahonda, España; Servicio de Obstetricia y Ginecología del Hospital Puerta de Hierro Majadahonda, Majadahonda, España

**Keywords:** cribado del primer trimestre para la trisomía 21, fracción beta libre de la gonadotropina coriónica humana, múltiplos de la mediana, proteína plasmática A asociada al embarazo, tasa de falsos positivos para la trisomía 21

## Abstract

**Objetivos:**

Existen diversos tipos de analizadores para realizar la cuantificación de los biomarcadores séricos de trisomía 21 empleados en el cribado del primer trimestre [fracción beta libre de la gonadotropina coriónica humana (β-hCG) y proteína plasmática A asociada al embarazo (PAPP-A)]. El objetivo del presente estudio es comparar la prestación analítica de cuatro analizadores diferentes y determinar sus respectivas tasas de falsos positivos (TFP) en la estimación del riesgo de trisomía 21.

**Métodos:**

Se llevó a cabo un estudio de seis meses de duración en el que se analizaron los datos de 1.136 mujeres gestantes, obtenidos en el cribado del primer trimestre. Cada muestra sérica fue procesada en cuatro analizadores distintos (IMMULITE 2000, Centaur XP, Cobas e-411, KRYPTOR compact PLUS), contando cada uno de ellos con su propio programa de estimación de riesgo. Posteriormente, se emplearon los métodos de Passing-Bablok y Bland-Altman para llevar a cabo un análisis comparativo de los valores obtenidos con cada analizador, tanto para los marcadores bioquímicos como para los múltiplos de la mediana (MoM).

**Resultados:**

Se observó una variabilidad significativa entre los distintos analizadores. En relación a la β-HCG libre, únicamente Centaur XP y KRYPTOR compact PLUS resultaron ser intercambiables. Con respecto a la PAPP-A, únicamente IMMULITE 2000 y Cobas e-411 arrojaron resultados comparables. No obstante, no se identificó ningún par de analizadores que pudiera considerarse intercambiable para la determinación simultánea de ambos marcadores. IMMULITE 2000 fue el analizador con los múltiplos de la mediana (MoM) de β-HCG libre más elevados (1.85 MoM (IQR: 1.4–2.97)) siendo el analizador KRYPTOR compact PLUS el que mostró valores más bajos (1.5 MoM (IQR: 1.23–2.21)). Los MoM de la PAPP-A más bajos se obtuvieron con el analizador IMMULITE 2000 (0.52 MoM (IQR: 0.38–0.82)) y los más altos se obtuvieron con el analizador Cobas e-411 (0.58 MoM (IQR: 0.39–0.90)). En la evaluación del riesgo, la totalidad de los analizadores detectaron todos los casos de T21 confirmados, aunque variaron en sus TFP, siendo Centaur XP (3.8 %), Cobas e-411 (2.5 %), y KRYPTOR compact PLUS (2.3 %) los que obtuvieron TFP inferiores al 5 %.

**Conclusiones:**

Para la cuantificación de los biomarcadores séricos del cribado del primer trimestre, ningún analizador es intercambable. KRYPTOR compact PLUS fue el analizador con la TFP más baja en la evaluación del riesgo de T21.

## Introducción

El cribado prenatal del primer trimestre para estimar el riesgo de presentar la trisomía 21 (T21), validado en 2009 por Nicolaides et al. [1], es una estrategia de elevada implantación en las consultas de obstetricia [2], [3], [4]. En esta prueba de cribado, se emplea un algoritmo que incluye tanto parámetros serológicos como ecográficos. Entre los biomarcadores séricos, se encuentran la fracción beta libre de la gonadotropina coriónica humana (β-hCG) y la proteína plasmática A asociada al embarazo (PAPP-A), siendo el grosor de translucencia nucal (TN) el parámetro ecográfico empleado [5]. Dichos parámetros son referidos a la edad gestacional, determinada mediante la medición ecográfica de la longitud cráneo caudal embrionaria.

Estos parámetros permiten la corrección del riesgo basal de trisomía 21, determinado inicialmente según la edad materna. A partir de dicho análisis, se puede clasificar a las gestantes como de alto o de bajo riesgo.

Si bien se ha demostrado que la prueba de ADN fetal libre (cffDNA) presenta mejores tasas de detección que las pruebas serológicas [6], esta técnica tiene un coste elevado, y no todos los laboratorios la realizan, presentando una relación coste-beneficio aceptable únicamente cuando se ofrece a gestantes de alto riesgo [7]. De este modo, el cribado del primer trimestre sigue siendo la primera opción para todas las gestantes [7], [8], [9], [10], [11], [12], realizándose un uso selectivo de la prueba de cffDNA sólo en pacientes con resultados positivos con el fin de minimizar los procedimientos invasivos.

En función de su nivel de riesgo, existen diversas estrategias para el manejo de las pacientes, aunque las guías clínicas coinciden en ofrecer una prueba diagnóstica (biopsia de vellosidades coriales (BVC) y amniocéntesis) confirmatoria en los casos de alto riesgo [7], [8], [9], [10], [11]. El inconveniente de estas pruebas invasivas es que pueden causar graves complicaciones, como un aborto espontáneo o infecciones, por lo que resulta esencial que la prueba de cribado inicial presente una tasa de falsos positivos (TFP) mínima, manteniendo a su vez una elevada tasa de detección. El cribado del primer trimestre han demostrado tener una tasa de detección del 80–90 %, con una TFP del 5 % [2], 9], 13], 14].

Actualmente, existen diversos analizadores automáticos para la cuantificación de la β-hCG libre y la PAPP-A en suero, contando todos ellos con su propio programa para calcular el riesgo de aneuploidía fetal. Estos dispositivos analíticos se basan en una serie de biomarcadores séricos, así como en el grosor de translucencia nucal (TN) y la edad materna, que conforman el punto de partida para evaluar el riesgo de T21. Paralelamente, se analizan otros parámetros como la edad gestacional, el peso materno, la raza, el tabaquismo, el tipo de concepción (natural o asistida) y el número de fetos [13], [15], [16], [17], [18]. Al ser intrínsecas de cada paciente, estas variables no dependen del dispositivo utilizado, a diferencia de los biomarcadores séricos, cuya medición sí está influida por la variabilidad analítica de cada analizador. En la estimación del riesgo, estas discrepancias entre analizadores se mitigan expresando los analitos β-HCG libre y PAPP-A como múltiplos de la mediana (MoM), en lugar de como unidades de concentración [19]. Estos MoM representan la desviación de una mediana, calculada a partir de datos de una población de referencia para cada edad gestacional. Sin embargo, cada dispositivo emplea como referencia bases de datos poblacionales distintos, con heterogeneidad en los orígenes, tamaño y composición de las poblaciones [20], hecho sobre el cual no es siempre informado el usuario.

La mencionada variabilidad entre los distintos analizadores puede resultar en discrepancias significativas en la estimación del riesgo [21], 22], lo que es motivo de preocupación, ya que la misma gestante puede obtener una clasificación de riesgo diferente, dependiendo del instrumento empleado. A pesar de ello, existen muy pocos estudios comparativos entre diferentes analizadores y su respectiva evaluación de riesgos, con resultados discrepantes. [22], [23], [24], [25]. A nuestro conocimiento, este es el primer estudio que se realiza para comparar la prestación analítica de cuatro analizadores y su TFP en el cribado del primer trimestre.

## Materiales y métodos

### Sujetos

Durante un periodo de seis meses (entre febrero y julio de 2020), se recogieron datos de 1.136 mujeres gestantes derivadas al Hospital Universitario Puerta de Hierro Majadahonda (España) para realizarles el cribado del primer trimestre. Las muestras séricas se obtuvieron entre las semanas 9 + 1 y 13 + 3 de gestación, mientras que el grosor de TN se determinó entre las semanas 10 + 6 y 13 + 6 de gestación [26].

Siguiendo la guía de práctica clínica actualmente empleada en nuestra comunidad autónoma (Madrid) [27], el cribado combinado del primer trimestre se realizó de forma secuencial, obteniéndose la muestra sérica y la medición de la translucencia nucal en semanas distintas de gestación. Un riesgo inferior a 1/270 se relacionó con un riesgo bajo de que el feto presentara T21, mientras que valores iguales o superiores a 1/270 se asociaron a un riesgo elevado. A aquellas gestantes identificadas como de alto riesgo se les ofreció la prueba de cffDNA. Si los resultados eran confirmatorios de un riesgo elevado, se las derivaba para realizarles una prueba invasiva (BVC o amniocentesis) que confirmara el diagnóstico. En caso de riesgo muy elevado (superior a 1/10), una TN por encima del percentil 99, o la detección ecocardiográfica de malformaciones, el protocolo recomendaba realizar directamente una prueba invasiva sin una prueba previa de cffDNA, aunque ninguna de las gestantes cumplió estos criterios.

Para calcular la tasa de falsos positivos (TFP), se utilizó un método en el que se tenían en cuenta el resultado del parto, el análisis genético posterior a la prueba diagnóstica (BVC o amniocentesis) o los resultados del estudio genético *post mortem* en los casos de aborto. De este modo, se clasificaron como negativos reales los neonatos no afectos de T21, aquellos con un resultado negativo en la prueba diagnóstica de T21, o con un resultado negativo para T21 en el estudio *post mortem*. Por otro lado, los neonatos afectos de T21, así como aquellos con un resultado positivo en la prueba diagnóstica de T21, o con un resultado positivo para T21 en el estudio *post mortem*, se clasificaron como positivos reales.

El estudio fue aprobado por el Comité de Ética del Hospital Puerta de Hierro Majadahonda (PI_13–20). Previo a la realización de las pruebas, se les proporcionó asesoramiento médico a las participantes, que otorgaron su consentimiento para la realización de los análisis genéticos.

### Muestras

En primer lugar, las 1.136 muestras séricas se procesaron empleando el analizador habitual, IMMULITE 2000 (Siemens Healthinneers, Erlangen, Alemania), para su análisis clínico. Posteriormente, las muestras se almacenaron a −80 °C durante seis meses. Tras dicho análisis, se estableció que 63 muestras presentaban un riesgo de T21≥1/270, por lo que fueron incluidas en el grupo de alto riesgo. El grupo de bajo riesgo estaba compuesto por 50 pacientes seleccionadas al azar de una muestra de 1.073 gestantes con un riesgo bajo (<1/270). Sin embargo, a cinco gestantes no se les pudo realizar la evaluación de riesgo debido a la falta de algunos datos ecocardiográficos, lo que resultó en una muestra final de 45 gestantes de bajo riesgo. La muestra total de pacientes incluidas en el presente estudio fue de 108 (n=108), divididas entre el grupo de alto riesgo (n=63) y bajo riesgo (n=45). Una vez seleccionadas las muestras del estudio, se descongelaron a temperatura ambiente y se homogeneizaron mediante agitación de vórtice.

### Instrumentos

Se realizó un estudio comparativo de cuatro instrumentos diferentes, entre los que se incluyeron: IMMULITE 2000, Centaur XP (Siemens Healthinneers), Cobas e-411 (Roche, Basel, Switzerland) y KRYPTOR compact PLUS (Thermo Fisher Scientific, Massachusetts, EE. UU). Tanto IMMULITE 2000 como Centaur XP emplean inmunoensayos de quimioluminiscencia, mientras que el analizador Cobas e-411 utiliza un inmunoensayo de electroquimioluminiscencia (ECLIA). Por otro lado, el dispositivo KRYPTOR Compact PLUS incluye una prueba de inmunofluorescencia automática basada en la tecnología TRACE *(Time-Resolved Amplified Cryptate Emission,* Emisión de Criptato Amplificada con Resolución Temporal).

El riesgo se evaluó en cada analizador empleando su programa correspondiente: PRISCA para IMMULITE 2000 y Centaur XP; SsdwLab6 para Cobas e-411; y Thermo Scientific Fast Screen™ para KRYPTOR compact PLUS.

Para calcular los MoM de la β-hCG libre y la PaPP-A, el programa PRISCA emplea datos de una cohorte de 842 gestantes caucásicas de primer trimestre recogidos en las pruebas rutinarias de cribado fetal en el Fetal Medicine Research Institute (Reino Unido). A lo largo del primer trimestre, se calcularon las medianas de la β-hCG y la PAPP-A al final de cada semana de gestación. Previamente al cálculo de las medianas, se excluyeron de la base de datos los embarazos gemelares y las gestantes con tabaquismo. Posteriormente, los MoM se actualizan periódicamente utilizando los datos generados en los cribados continuos de laboratorio. En el momento de este estudio, los MoM se calcularon a partir de los datos históricos del laboratorio.

Con respecto al programa SsdwLab6, los MoM se calcularon utilizando los datos de un estudio que proporcionaba 4.746 valores de β-hCG libre y PAPP-A de gestantes de primer trimestre (entre las semanas gestacionales 8+0 y 13+6). A partir de la regresión de las medianas calculadas por día, se estimaron los valores medianos diarios para cada edad gestacional [28].

Fast Screen™ emplea las medianas obtenidas en un estudio realizado por Wright y col. que incluía muestras séricas de 222.475 gestantes, entre las que se encontraban 886 gestaciones afectas de trisomía 21 [26].

Todos los programas utilizaban los mismos conjuntos de datos, que incluían valores de β-hCG libre y PAPP-A, paralelamente a la edad gestacional, edad materna, peso materno, tabaquismo, presencia de diabetes, raza, tipo de concepción (natural frente a *in vitro*), y el número de fetos. La totalidad de los parámetros se mantuvo constante, salvo los valores de β-hCG libre y PAPP-A, que variaron en función del instrumento empleado y las medianas de referencia empleadas por cada programa. En los cuatro métodos, los valores de β-hCG libre se estandarizaron aplicando el patrón de Preparación Internacional de Referencia para la subunidad β de la gonadotropina coriónica humana del National Institute for Biological Standards and Control (NIBSC) (código 75/551). Por otro lado, en los analizadores Cobas e-411 y KRYPTOR Compact PLUS, la cuantificación de la PAPP-A se realizó siguiendo el patrón de Preparación Internacional de Referencia de la OMS (IRP 78/610). En los analizadores IMMULITE 2000 y Centaur XP, la determinación de la PAPP-A se realizó aplicando un patrón interno elaborado con materiales y procedimientos de medición validados, tal como se indica en los folletos técnicos explicativos.

### Análisis estadístico

Siguiendo las recomendaciones EP09-A3 del CLSI, aplicamos los métodos de Passing-Bablok y Bland-Altman para comparar las concentraciones y los MoM de los marcadores bioquímicos séricos obtenidos con los distintos analizadores [29]. La regresión de Passing-Bablok descartó la intercambiabilidad de los resultados obtenidos con los distintos analizadores si se aplicaba alguno o varios de los siguientes criterios: si el intervalo de confianza del 95 % (IC95 %) de la ordenada en el origen no incluía el valor cero (0) y/o el IC95 % de la gradiente no incluía el valor uno (1) [30]. En el análisis de Bland-Altman, se identificó un sesgo significativo cuando la línea de igualdad (0) no se encontraba dentro del IC del 95 % de la diferencia media (p<0.05) [31]. Los resultados de los instrumentos y programas de evaluación de riesgo únicamente se consideraron intercambiables si ninguna de las dos técnicas (Passing-Bablok y Bland-Altman) revelaba un sesgo significativo. La normalidad de los datos se evaluó empleando la prueba de Shapiro-Wilk. Un valor p<0.05 se consideró estadísticamente significativo.

Para el análisis estadístico, se empleó el programa MedCalc Statistical Software versión 18.2.1 (MedCalc Software bvba, Ostend, Bélgica, http://www.medcalc.org; 2018).

## Resultados

### Comparación de los biomarcadores

Las concentraciones de β-hCG libre y PAPP-A se expresaron en ng/mL y mIU/mL, respectivamente. En el análisis de β-hCG libre, los análisis de regresión realizados para analizar la prestación analítica de los analizadores revelaron coeficientes de correlación de Spearman (ρ) superiores a 0.97 en todos los casos. Sin embargo, salvo al comparar Centaur XP con KRYPTOR compact PLUS y con Cobas e-411, el resto de analizadores mostraron un error proporcional, dado que ninguno de ellos cumplía el criterio establecido para la pendiente. Asimismo, Cobas e-411 mostró un error constante al compararlo con Centaur XP y con KRYPTOR compact PLUS, pero no con IMMULITE 2000. El análisis de Bland-Altman indicó un sesgo significativo (p<0.001) en todas las comparaciones, excepto al comparar Centaur XP con KRYPTOR compact PLUS (p=0.389). De este modo, únicamente los resultados de Centaur XP y KRYPTOR compact PLUS resultaron ser intercambiables según los análisis de Passing-Bablok y Bland-Altman.

Con respecto a la cuantificación de PAPP-A, el análisis de regresión mostró que solo los resultados de IMMULITE 2000 y Cobas e-411 eran comparables, mientras que el resto de comparaciones revelaron un error constante. Del mismo modo, estos dos analizadores fueron los únicos que no mostraron diferencias significativas en la cuantificación de la PAPP-A en el análisis de Bland-Altman (p=0.836).

En la Tabla 1 se detallan los resultados de las comparaciones entre los distintos analizadores. En las Figuras Suplementarias 1 y 2 se muestran los diagramas de dispersión de los análisis de Passing-Bablok relativos a los valores de β-hCG libre y PAPP-A, respectivamente. Los análisis de Bland-Altman están representados en las Figuras Suplementarias 3 y 4.

**Tabla 1:** Comparación de los niveles de β-hCG libre y PAPP-A entre los diferentes analizadores.

Analizadores	Analito	ρ	Ordenada (IC95%)	Pendiente (IC95 %)	Media (IC95 %)	p Valor
IMMULITE 2000 vs. Centaur XP	Β-hCG libre	0,988	0,17 (−1,63a 1,53)	0,87 (0,84–0,90)^a^	15,9 (10,9 a 20,8)^a^	<0,001
	PAPP-A	0,979	0,02 (−0,03 a 0,06)	1,48 (1,42–1,54)^a^	−0,69 (−0,8 a −0,58)^a^	<0,001
IMMULITE 2000 vs. KRYPTOR compact PLUS	Β-hCG libre	0,981	2,12 (−0,33 a 3,71)	0,85 (0,82–0,89) ^a^	17,3 (13,1 a 21,6)^a^	<0,001
	PAPP-A	0,978	0,004 (−0,03 a 0,04)	1,21 (1,18–1,25)^a^	−0,34 (−0,41 a −0,28)^a^	<0,001
IMMULITE 2000 vs. Cobas e-411	Β-hCG libre	0,978	−1,63 (−4,05 a 0,40)	0,83 (0,79–0,86)^a^	20,7 (16,5 a 24,9)^a^	<0,001
	PAPP-A	0,973	0,03 (−0,01 a 0,07)	0,96 (0,92–1,01)	−0,005 (−0,05 a 0,04)	0,836
Centaur XP vs. KRYPTOR compact PLUS	Β-hCG libre	0,985	1,32 (−0,74 a 3,45)	0,97 (0,94–1,02)	1,17 (−1,51 a 3,86)	0,389
	PAPP-A	0,994	0,02 (−0,01 a 0,03)	1,21 (1,18–1,24)^a^	0,35 (0,28 a 0,4)^a^	<0,001
Centaur XP vs. Cobas e-411	Β-hCG libre	0,98	−2,3 (−4,78 a 0,76)^a^	0,97 (0,93–1,01)	5,94 (3,75 a 8,13)^a^	<0,001
	PAPP-A	0,988	0,03 (−0,01 a 0,05)	0,66 (0,64–0,69)^a^	0,69 (0,57 a 0,8)^a^	<0,001
KRYPTOR compact PLUS vs. Cobas e-411	Β-hCG libre	0,969	3,52 (1,21 a 6,06)^a^	1,02 (0,97–1,07)	4,83 (2,23 a 7,53)^a^	<0,001
	PAPP-A	0,989	0,04 (0,01 a 0,67)^a^	0,79 (0,76–0,81)^a^	0,34 (0,27 a 0,41)^a^	<0,001

ρ, coeficiente de correlación de Spearman; IC95 %, intervalo de confianza del 95 %. ^a^No se cumplen los criterios de intercambiabilidad aceptable.

### Comparación de MoM

En términos generales, se obtuvieron coeficientes ρ más bajos para los MoM que para los analitos. Así, a diferencia de la comparación de los analitos, el análisis comparativo de los MoM descartó que los MoM obtenidos por los diferentes instrumentos para la β-hCG libre fueran intercambiables, ya que todos mostraron sesgos en uno o en ambos análisis estadísticos. Únicamente IMMULITE 2000 y Cobas e-411 mostraron un resultado aceptable en el análisis de Passing-Bablok, pero no en el análisis de Bland-Altman. Al comparar los MoM de la β-hCG libre, el análisis de Bland-Altman reveló diferencias significativas en todos los casos, salvo en Centaur XP y Cobas e-411, que únicamente mostraron sesgo en la regresión de Passing-Bablok. A continuación se muestran las medianas de los MoM de la β-hCG libre obtenidas para los distintos instrumentos: 1.85 MoM (IQR: 1.4–2.97) con IMMULITE 2000, 1*.*75 MoM (IQR: 1.28–2*.*54) con Centaur XP, 1.75 MoM (IQR: 1.27–2.62) con Cobas e-411, and 1.5 MoM (IQR: 1.23–2.21) con KRYPTOR compact PLUS.

El análisis de regresión de los MoM de la PAPP-A entre los diferentes pares de instrumentos mostró que todos cumplían los criterios establecidos, a excepción de IMMULITE 2000 frente a KRYPTOR compact PLUS, que mostraron un sesgo constante. El análisis de Bland-Altman reveló un valor p<0.001 para todas las comparaciones en las que se incluía el analizador IMMULITE 2000. Por el contrario, el resto de analizadores no revelaron significación estadística, con valores p de 0.96 al comparar Centaur XP con KRYPTOR compact PLUS; 0.09 en la comparación de Centaur XP frente a Cobas e-411; y 0.24 para KRYPTOR compact PLUS frente a Cobas e-411. Con respecto a las medianas, la mediana de los MoM de la PAPP-A para IMMULITE 2000 fue de 0.52 (IQR: 0.38–0.82); 0.55 (IQR: 0.41–0.91) para Centaur XP; 0.54 (IQR: 0.42–0.84) para KRYPTOR compact PLUS; y 0.58 (IQR: 0.39–0.90) para Cobas e-411.

En la Tabla 2 se muestra un resumen de los resultados del estudio. Los diagramas de dispersión de la β-hCG libre y la PAPP-A obtenidos con el método de Passing-Bablok se muestran en las Figuras Suplementarias 5 y 6, mientras que los correspondientes al método Bland-Altman se han incluido en las Figuras Suplementarias 7 y 8.

**Tabla 2:** Comparación de los MoM de la β-hCG libre y de la PAPP-A entre los diferentes programas.

Analizadores	MoM	ρ	Ordenada (IC95 %)	Pendiente (IC95 %)	Media (IC95 %)	p Valor
IMMULITE 2000 vs. Centaur XP	Β-hCG libre	0,973	0,11 (0,01 a 0,19)^a^	0,87 (0,82–0,92)^a^	0,19 (0,11a 0,29)^a^	<0,001
	PAPP-A	0,957	0,04 (−0,02 a 0,06)	1,04 (0,98–1,13)	−0,09 (−0,13 a −0,05)^a^	<0,001
IMMULITE 2000 vs. KRYPTOR compact PLUS	Β-hCG libre	0,901	0,03 (−0,09 a 0,16)	0,81 (0,75–0,88)^a^	0,43 (0,31 a 0,55)^a^	<0,001
	PAPP-A	0,935	0,03 (0,002 a 0,08)^a^	1,02 (0,92–1,08)	−0,08 (−0,13 a −0,04)^a^	<0,001
IMMULITE 2000 vs. Cobas e-411	Β-hCG libre	0,91	−0,05 (−0,25 a 0,81)	0,98 (0,89–1,05)	0,24 (0,09 a 0,38)^a^	0,002
	PAPP-A	0,882	−0,01 (−0,06 a 0,04)	1,03 (0,95–1,13)	−0,06 (−0,10 a −0,01)^a^	0,015
Centaur XP vs. KRYPTOR compact PLUS	Β-hCG libre	0,969	−0,003 (−0,1 a 0,12)	0,9 (0,84–0,97)^a^	0,19 (0,11 a 0,27)^a^	<0,001
	PAPP-A	0,952	0,01 (−0,03 a 0,04)	1,01 (0,94–1,08)	0,0 (−0,03 a 0,03)	0,959
Centaur XP vs. Cobas e-411	Β-hCG libre	0,958	−0,19 (−0,37 a −0,03)^a^	1,13 (1,02–1,23)^a^	−0,01 (−0,13 a 0,12)	0,924
	PAPP-A	0,973	−0,01 (−0,04 a 0,02)	0,95 (0,9–1,01)	0,03 (−0,004 a 0,06)	0,085
KRYPTOR compact PLUS vs. Cobas e-411	Β-hCG libre	0,947	−0,13 (−0,31 a −0,001)^a^	1,2 (1,09–1,32)^a^	−0,19 (−0,31 a −0,08)^a^	0,002
	PAPP-A	0,947	−0,02 (−0,09 a 0,03)	1 (0,91–1,12)	0,03 (−0,02 a 0,07)	0,235

MoM, múltiplos de la mediana; ρ, coeficiente de correlación de Spearman; IC95 %, intervalode confianza del 95 %. ^a^No se cumplen los criterios de intercambiabilidad aceptable.

### Estimación de riesgo

Todas las gestantes con un riesgo≥1/270 (n=63) (determinado con IMMULITE 2000) fueron derivadas para realizarles a un análisis de cffDNA. De estas, 58 resultaron presentar un riesgo bajo, 56 de las cuales tuvieron resultados de parto normales, incluyendo a una paciente con parto gemelar. Dos de las gestantes con bajo riesgo según el análisis de cffDNA sufrieron un aborto espontáneo, habiéndose descartado anomalías en el análisis de ADN *post mortem*. De las restantes cinco gestantes con riesgo elevado, el análisis de cffDNA identificó la presencia de T21 en la totalidad de los casos. Una vez confirmado el resultado, se sometió a las cinco pacientes a una técnica invasiva (3 amniocentesis y 2 BVC), lo que derivó en el diagnóstico de T21 en cuatro de los casos. Posteriormente, las gestantes optaron por la interrupción del embarazo. En la quinta paciente, la BVC reveló un resultado genético normal, pero esta sufrió un aborto espontáneo asociado a la realización del procedimiento invasivo. Cabe señalar que el análisis de la muestra en los otros tres analizadores también reveló un riesgo elevado, por lo que la paciente hubiera sido derivada igualmente para la realización de una prueba invasiva. Las pacientes con un riesgo de T21 inferior a 1/270 dieron a luz a niños y niñas euploides, salvo en un caso en el que se produjo un aborto espontáneo. El análisis de ADN realizado al feto descartó la presencia de trisomía.

En cuanto a la prestación analítica, todos los analizadores identificaron correctamente los cuatro casos de T21 como casos de alto riesgo, lo que se traduce en una tasa de falsos negativos del 0 % en nuestro estudio de cohorte. Con respecto a los falsos positivos, IMMULITE 2000 identificó a 63 de las 1.137 gestantes como casos de alto riesgo. De estas, cuatro fueron positivos reales, lo que resulta en 59 falsos positivos, lo que arroja una tasa de falsos positivos (TFP) del 5.2 % (59/1.137). Centaur XP mostró una tasa de falsos positivos del 3.8 % (43/1.137), mientras que Cobas arrojó una tasa de falsos positivos del 2.5 % (28/1.137), y KRYPTOR compact PLUS del 2.3 % (26/1.137).

## Discusión

El cribado de la trisomía 21 en gestantes cuenta con una amplia implantación, utilizándose para realizarla diversos métodos. En los últimos años, se ha demostrado la superioridad del análisis de cffDNA sobre los otros métodos, aunque su elevado coste ha llevado a restringir su uso a las gestantes con un riesgo elevado de T21 [6], 7]. En relación al cribado de muestras séricas, el método de mayor prevalencia es el cribado del primer trimestre, en el cual se calcula el riesgo de T21 a partir de parámetros tanto clínicos (grosor de TN) como bioquímicos (β-hCG libre y PAPP-A) [2]. Para tener en cuenta la variabilidad de los analitos bioquímicos entre las diferentes edades gestacionales, se realiza una conversión de los valores a múltiplos de la mediana (MoM) para calcular el riesgo de T21. Para la cuantificación de estos analitos, existen diversos instrumentos, cada uno de los cuales emplea sus propios MoM. El presente estudio se llevó a cabo para evaluar la intercambiabilidad de los resultados de β-hCG libre y PAPP-A y sus MoM entre los diferentes analizadores.

La comparación de los marcadores bioquímicos reveló que únicamente eran intercambiables los resultados de β-hCG libre obtenidos con Centaur XP y KRYPTOR compact PLUS, y los valores de PAPP-A obtenidos con IMMULITE 2000 y Cobas e-411. No obstante, ninguno de los instrumentos resultó ser intercambiable simultáneamente para ambos analitos. Dado que los dos analitos son necesarios para calcular el riesgo, concluimos que ningún analizador puede ser sustituido por otro para realizar la cuantificación de la β-hCG libre y la PAPP-A.

Con respecto a los MoM, IMMULITE 2000 fue el que arrojó MoM de la β-hCG libre más altos, frente a los otros instrumentos. Centaur XP y Cobas e-411 mostraron MoM de la β-hCG libre comparables, mientras que los valores más bajos de MoM se obtuvieron con KRYPTOR compact PLUS. Con respecto a los MoM de la PAPP-A, IMMULITE 2000 arrojó los valores más bajos, siendo los obtenidos con Cobas e-411 los más altos. Centaur XP y KRYPTOR compact PLUS mostraron medianas intermedias de MoM de la PAPP-A comparables. Engell y col [23] compararon los MoMs de β-hCG y PAPP-A obtenidos con Cobas e-411 y KYRPTOR, concluyendo que Cobas e-411 muestra valores más bajos que los obtenidos con KRYPTOR compact PLUS. Por otro lado, Hörmansdörfer y col [24] observaron que se obtenían valores de MoM de PAPP-A más bajos con el analizador KRYPTOR compact PLUS que con Cobas e-411, pero resultados similares en los MoM de la β-hCG con los dos instrumentos. Nuestros hallazgos coinciden con los de este último estudio, ya que obtuvimos valores de MoM de PAPP-A más altos con Cobas e-411 que con KRYPTOR compact PLUS. En relación a los MoM de β-hCG libre, observamos una variación de 0.2 entre las medianas de los dos instrumentos, mientras que Hörmansdörfer y col. únicamente hallaron una diferencia de 0.02 entre las medianas de los MoM. Así mismo, en otro estudio [25] se compararon los analizadores Cobas e-411 e IMMULITE 2000, concluyendo que existía una correlación significativa entre ambos, una conclusión que no pudimos replicar. Los análisis de Passing-Bablok y Bland Altman revelaron la ausencia de sesgo únicamente al comparar los MoM de la PAPP-A obtenidos con Centaur XP y KRYPTOR compact PLUS, y con Centaur XP y Cobas e-411, respectivamente. Cabe señalar que estos hallazgos no son extensibles a los MoM de la β-hCG libre, que presentaron un sesgo significativo en todas las comparaciones entre analizadores. En consecuencia, concluimos que ninguno de los analizadores es intercambiable, por lo que cada uno de ellos debe emplear su propio programa de evaluación de riesgo.

Teniendo en cuenta el número de casos analizados, la diferencia más notable entre los analizadores se observó en sus tasas de falsos positivos. Con respecto a las estimaciones de riesgo, todos los analizadores detectaron la totalidad de los casos positivos, aunque únicamente Centaur XP, Cobas e-411 y KRYPTOR compact PLUS alcanzaron una TFP apropiada inferior al 5 % [32], [33], [34], [35], siendo KRYPTOR compact PLUS el que obtuvo una tasa más baja. Cabe mencionar que, aunque IMMULITE 2000 y Centaur XP emplean el mismo programa de estimación de riesgo y los mismos MoM, estos dispositivos mostraron diferentes tasas de falsos positivos (5.2 % y 3.8 %, respectivamente), lo que indica diferencias en la cuantificación de los analitos. Otro estudio comparativo entre KRYPTOR compact PLUS e IMMULITE 2000 reveló un mejor rendimiento de KRYPTOR compact PLUS, lo que coincide con nuestros hallazgos [22]. Asimismo, Engell y col [23]. observaron variaciones en los MoM, aunque no hallaron diferencias en la prestación analítica general de KRYPTOR compact PLUS y Cobas e-411, que fue del 5.1 % para ambos. Sin embargo, las discrepancias entre nuestra tasa de falsos positivos y la suya podría atribuirse a diferencias en el programa de evaluación del riesgo. Las variaciones en los MoM entre los diferentes analizadores indican diferencias en las medianas empleadas por cada programa para realizar dicho cálculo. Estas discrepancias surgen de los datos poblacionales empleados para establecer dichas medianas, hecho que, en algunos casos, el usuario desconoce. Aunque optamos por emplear los programas asociados a cada analizador suministrados por ThermoFisher y Roche, respectivamente, Engell y col [23]. emplearon el mismo programa (Astraia, Gmbh, Munich, Alemania) para ambos instrumentos, que no se correspondía con el suministrado por ninguno de los fabricantes.

La principal limitación de este estudio radica en el relativamente bajo número de positivos reales. El presente estudio comparativo se llevó a cabo durante un periodo de transición en el uso de instrumentos analíticos. Dado que se nos instó a seleccionar la nueva plataforma que mejor se adaptara a nuestras necesidades en un periodo de tiempo limitado, ampliar el periodo de recogida de muestras no fue posible, por lo que no pudimos reunir más casos de positivos reales. Por otro lado, dada la situación de competición interna, ninguno de los instrumentos se sometió a un control externo.

En cuanto al cffDNA, de todas las pacientes de alto riesgo identificadas mediante el cribado del primer trimestre, la prueba de cffDNA identificó correctamente todos los casos de alto riesgo, que fueron posteriormente confirmados mediante una técnica invasiva. No obstante, en un caso, la prueba de cffDNA detectó una T21 que no fue posteriormente corroborada por la BVC. En los casos restantes, los resultados del análisis de cffDNA fueron negativos, lo que se confirmó tras el parto. Estos hallazgos muestran la elevada, que no infalible, precisión de la prueba de cffDNA, aunque el cribado del primer trimestre para T21 continúa siendo el método de elección en España, debido a su mayor accesibilidad. Aun cuando el análisis de cffDNA esté disponible, se trata de una técnica con un coste elevado, por lo que resulta preciso que la tasa de falsos positivos sea baja. Lo contrario resultaría en un incremento innecesario del gasto sanitario, tensionando así el sistema, al requerir la realización de pruebas confirmatorias y procedimientos de seguimiento adicionales. Esto no solo comprometería la eficiencia del sistema, sino que también sumaría presión a las pacientes, que recibirían un resultado patológico provisional que les provocaría ansiedad y estrés emocional.

En conclusión, los analizadores IMMULITE 2000, Centaur XP, KRYPTOR compact PLUS, y Cobas e-411 no son intercambiables. De este modo, en el caso de que se sustituyera el analizador que se empleó en el cribado del primer trimestre para T21, el nuevo instrumento deberá contar con su propio programa de evaluación de riesgo, debiendo dicho cambio ser debidamente comunicado al personal clínico. Por último, uno de los principales aspectos a tener en cuenta a la hora de seleccionar un analizador es su tasa de falsos positivos, con el objeto de minimizar las derivaciones innecesarias para la realización de técnicas invasivas.

## Supplementary Material

Supplementary Material

## Material Suplementario

Este artículo incluye material suplementario (https://doi.org/10.1515/almed-2025-0149).
