# Supplementary material for: Estudio comparativo de cuatro analizadores diferentes para la evaluación prenatal del riesgo de trisomía 21
Source: Adv Lab Med. 2025 Nov 11;6(4):419–26. [Article in Spanish] doi: 10.1515/almed-2025-0149 (PMC12744382; doi:10.1515/almed-2025-0149)

**Figura Suplementaria 1.** Diagramas de dispersión de Passing-Bablok para los niveles de β-HCG libre.


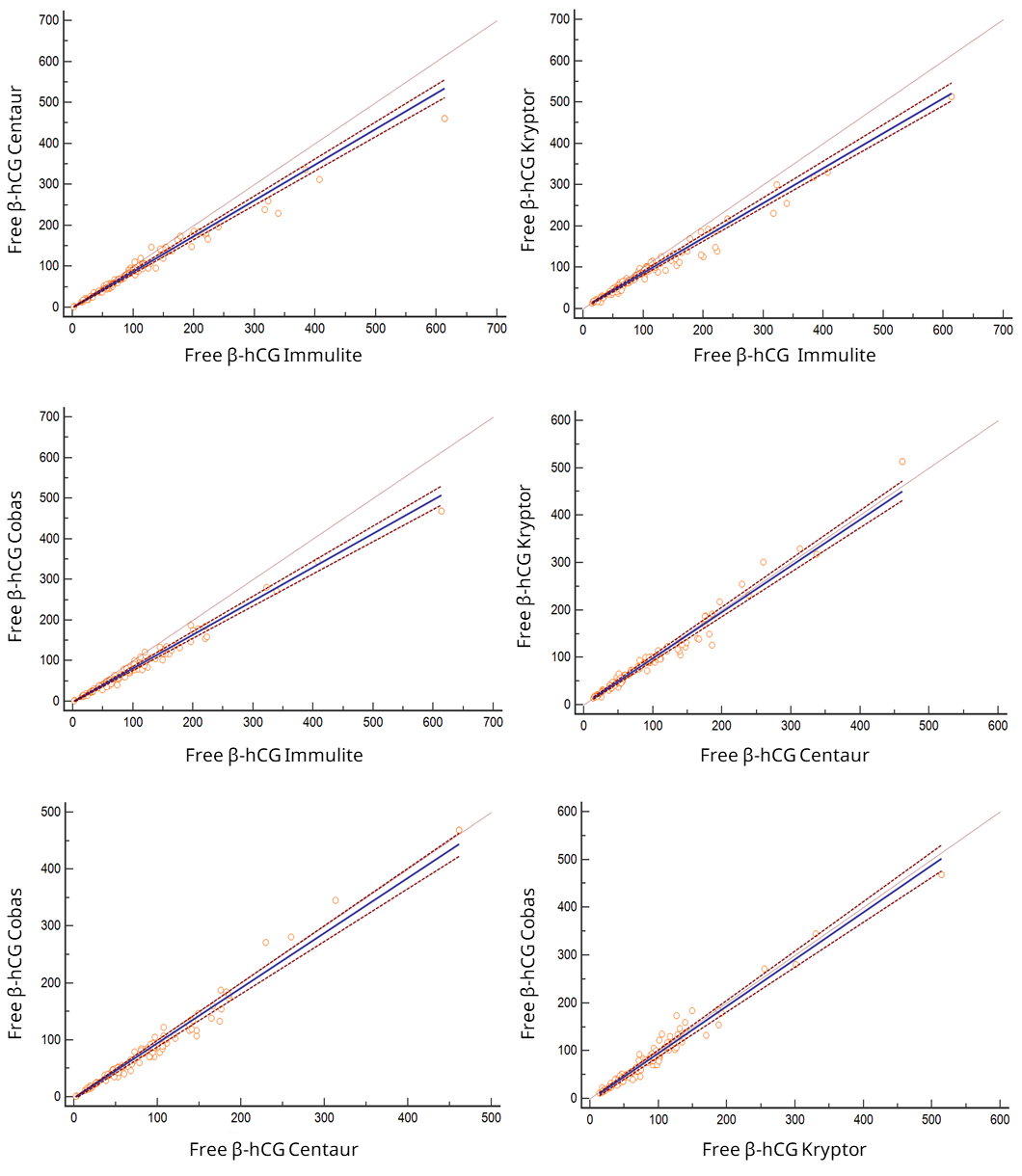


**Figura Suplementaria 2.** Diagramas de dispersión de Passing-Bablok para los niveles de PAPP-A.


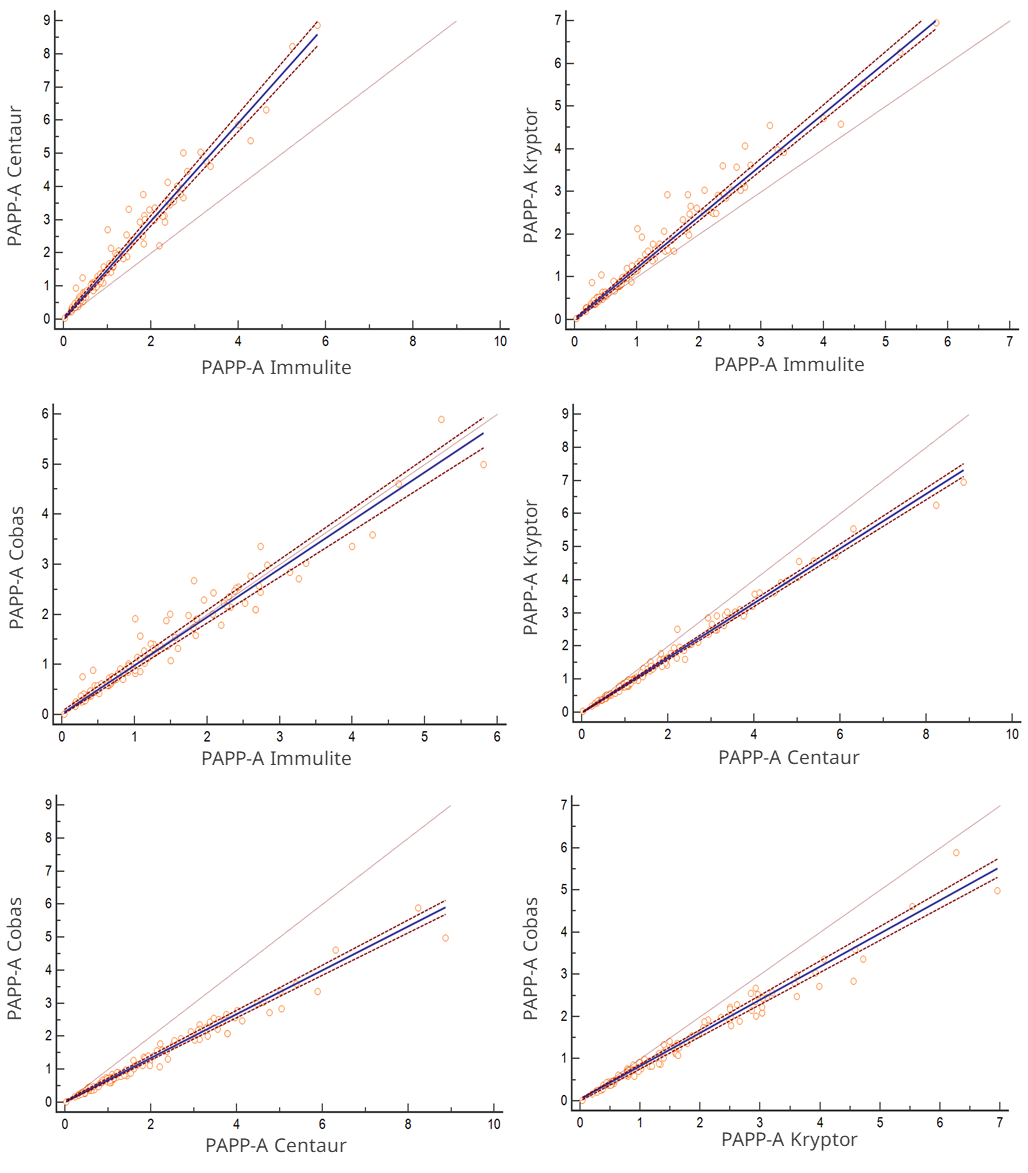


**Figura Suplementaria 3**. Diagramas de dispersión de Bland-Altman para los niveles de β-HCG libre.


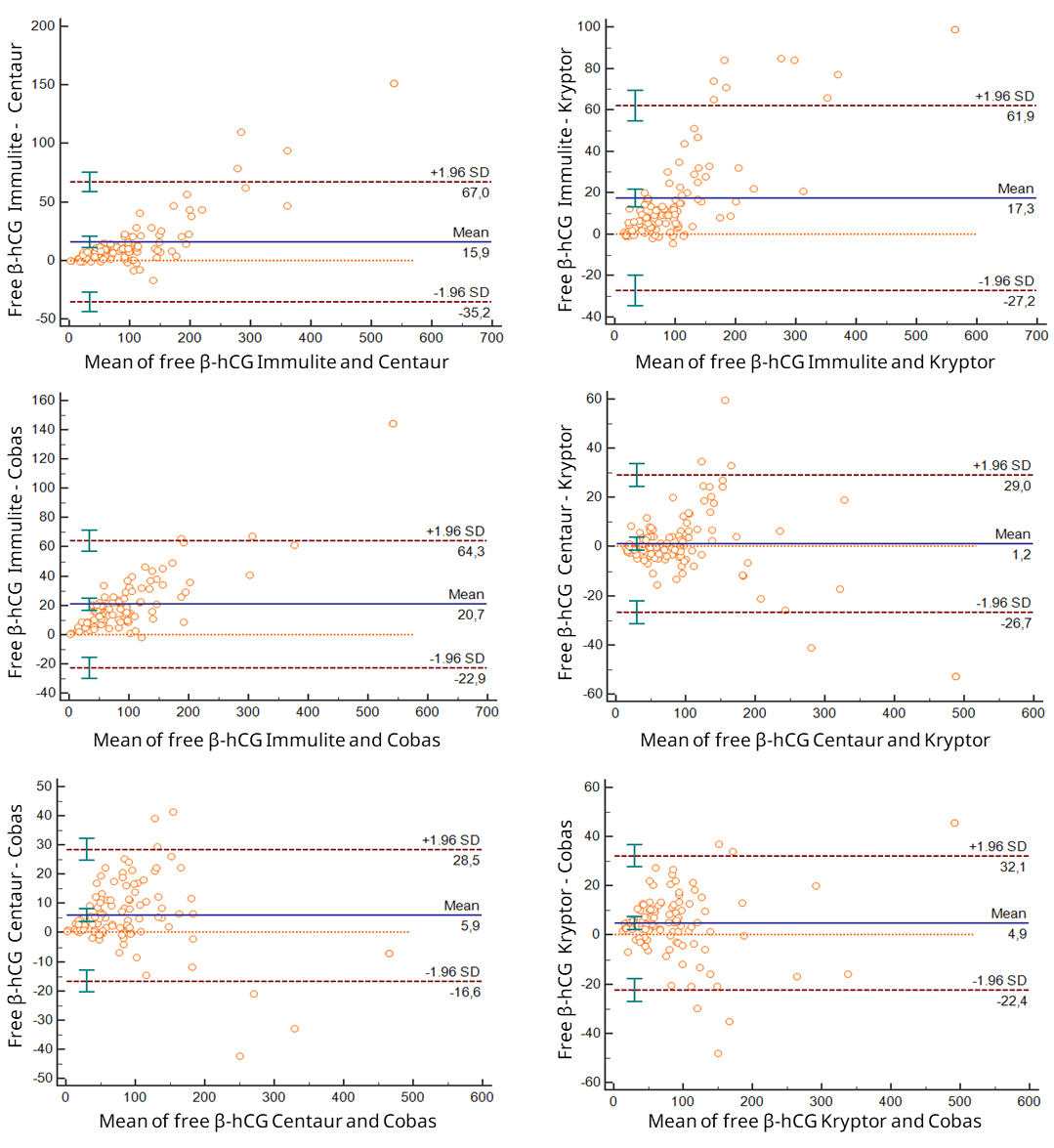


**Figura Suplementaria 4.** Diagramas de dispersión de Bland-Altman para los niveles de PAPP-A.


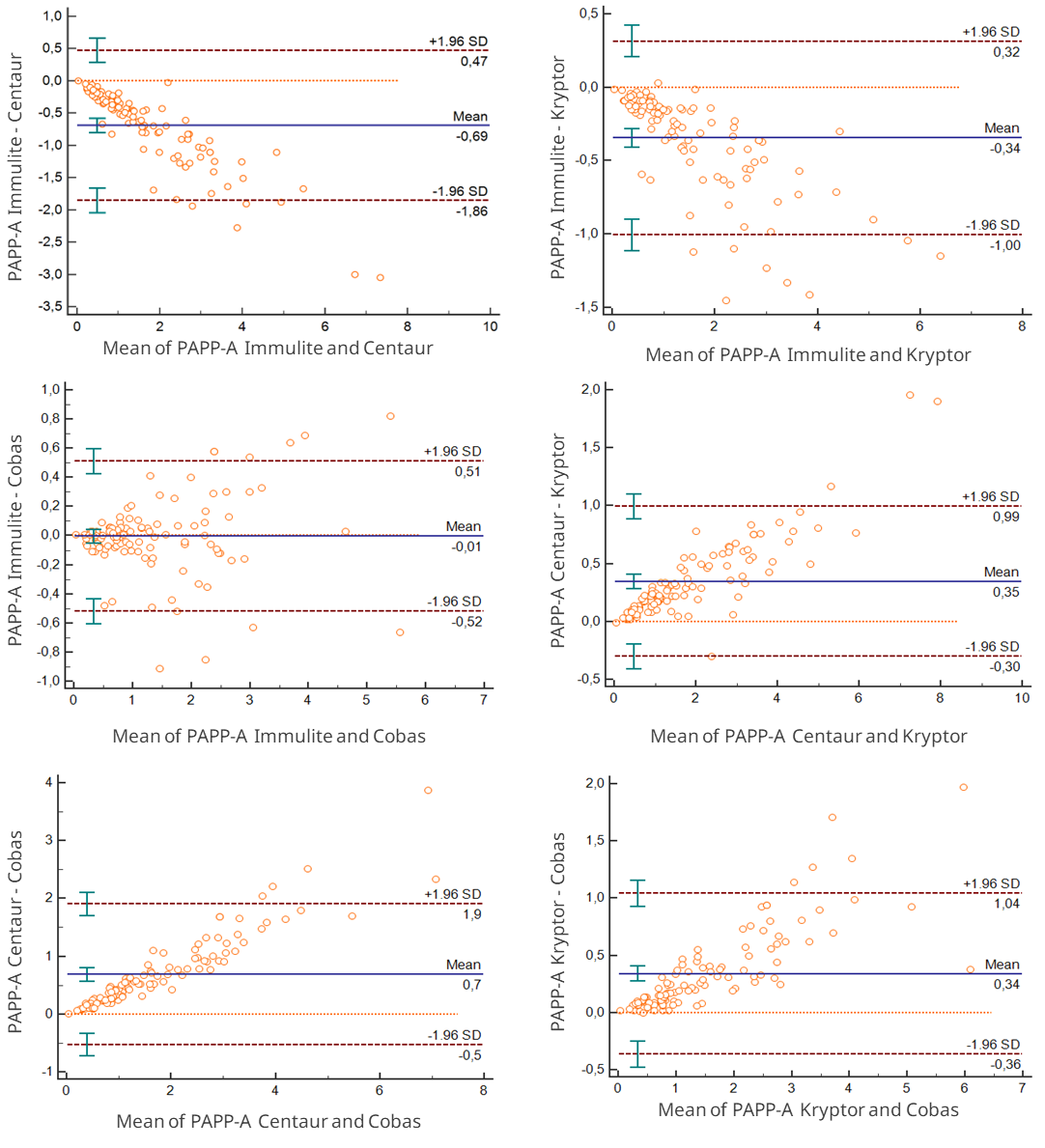


**Figura Suplementaria 5.** Diagramas de dispersión de Passing-Bablok para los MoM de β-HCG libre.


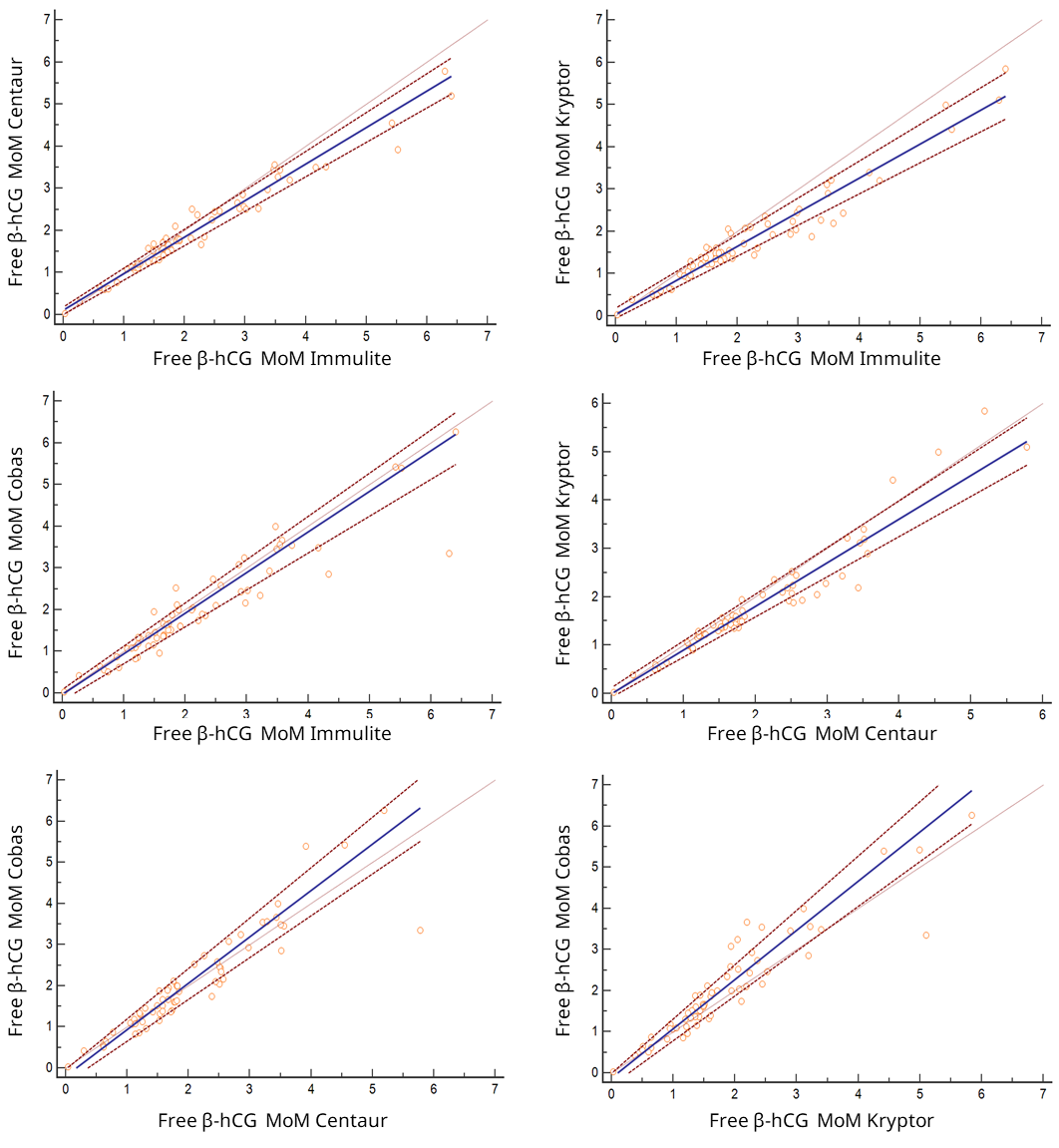


**Figura Suplementaria 6**. Diagramas de dispersión de Passing-Bablok para los MoM de la PAPP-A.


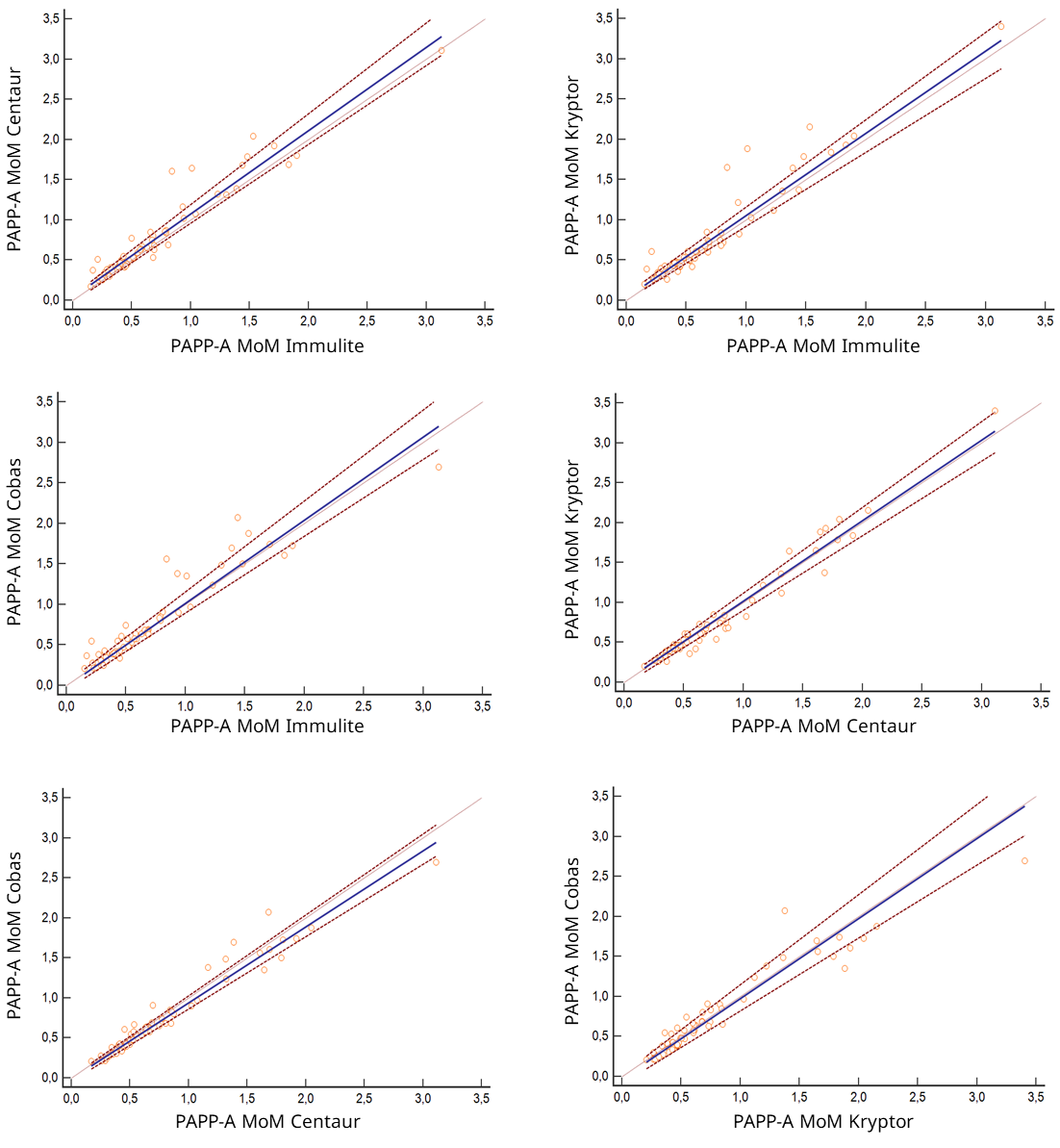


**Figura Suplementaria 7.** Diagramas de dispersión de Bland-Altman para los MoM de la β-HCG libre.


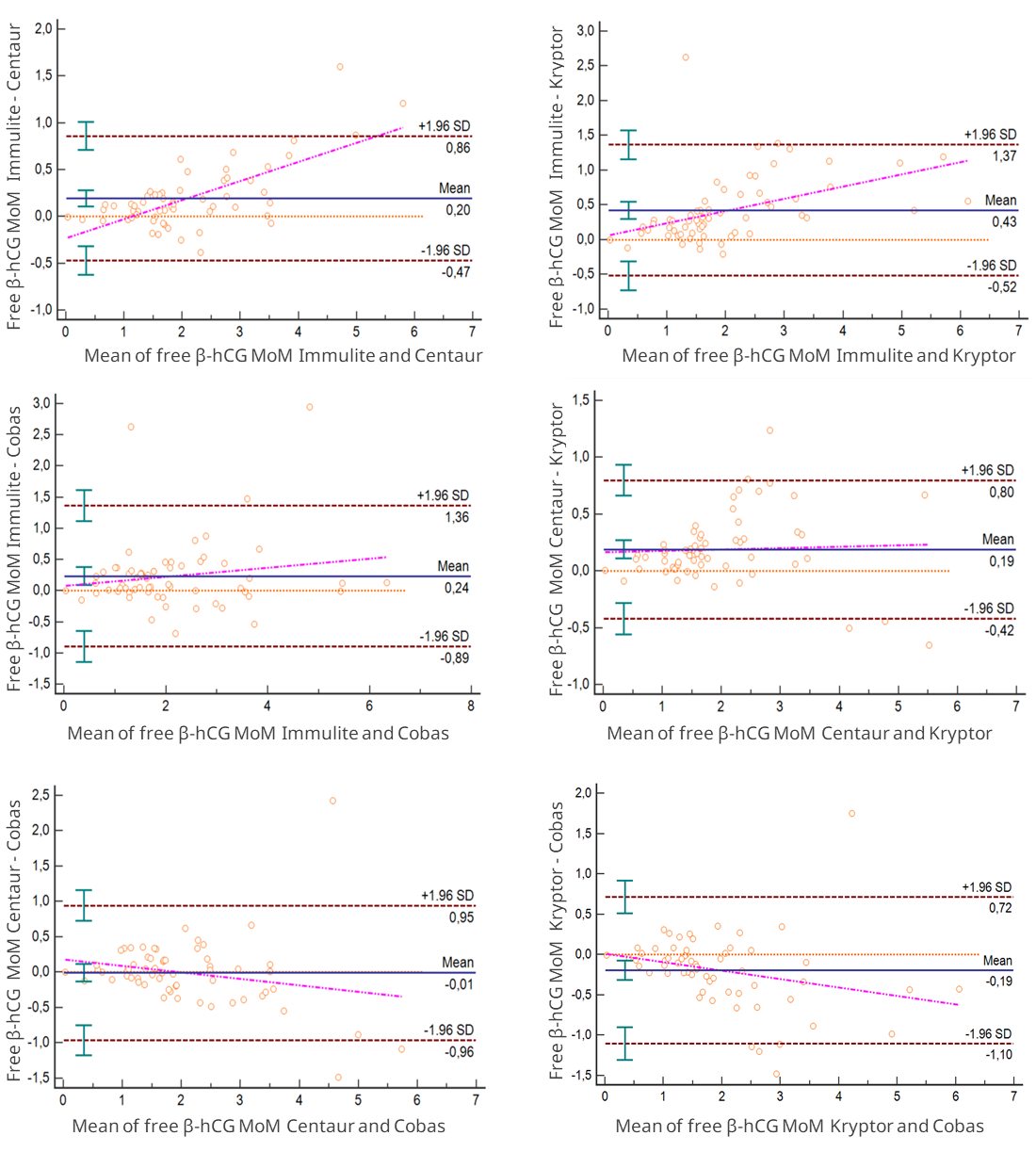


**Figura Suplementaria 8.** Diagramas de dispersión de Bland-Altman para los MoM de la PAPP-A.


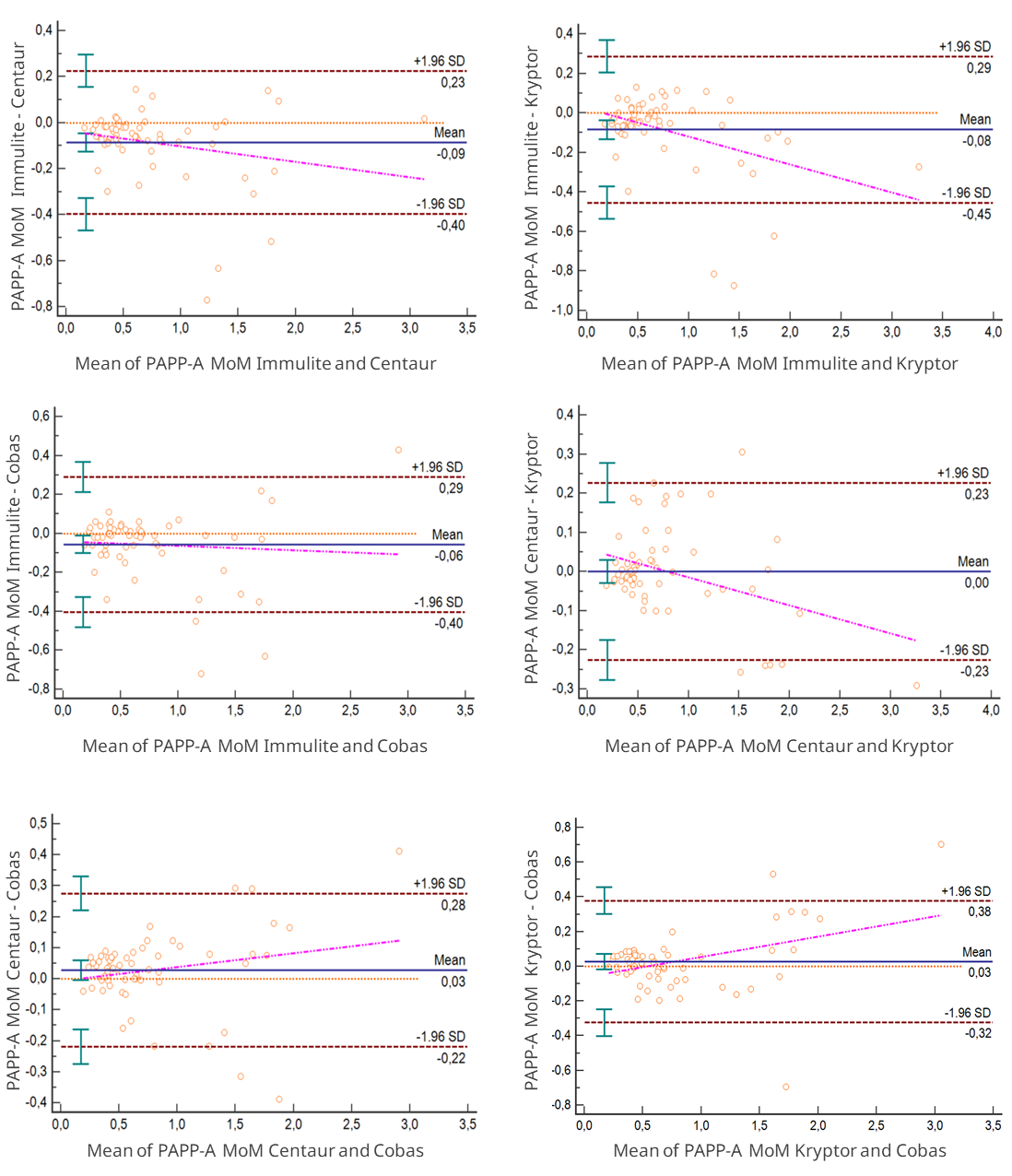

Supplement: Supplementary file 1 — Supplementary Material [file j_almed-2025-0149_suppl_001.docx]
